# Physiological and Psychological Effects of a Walk in Urban Parks in Fall

**DOI:** 10.3390/ijerph121114216

**Published:** 2015-11-09

**Authors:** Chorong Song, Harumi Ikei, Miho Igarashi, Michiko Takagaki, Yoshifumi Miyazaki

**Affiliations:** 1 Center for Environment, Health and Field Sciences, Chiba University, 6-2-1 Kashiwa-no-ha, Kashiwa, Chiba 277-0882, Japan; E-Mails: crsong1028@chiba-u.jp (C.S.); ikei0224@ffpri.affrc.go.jp (H.I.); miho.murachi@gmail.com (M.I.); mtgaki@faculty.chiba-u.jp (M.T.); 2 Forestry and Forest Products Research Institute, 1 Matsunosato, Tsukuba, Ibaraki 305-8687, Japan

**Keywords:** urban green space, relaxation, heart rate, heart rate variability, profile of mood states, state-trait anxiety inventory

## Abstract

In recent times, attention has been focused on the role of urban green spaces in promoting human health and well-being. However, there is a lack of evidence-based research on the physiological effects of walking in urban green areas. This study aimed to clarify the physiological and psychological effects of walking in urban parks during fall. Twenty-three males (mean age 22.3 ± 1.2 years) were instructed to walk predetermined 15-min courses in an urban park and in a nearby city area (control). Heart rate and heart rate variability were measured to assess physiological responses, and the semantic differential method, Profile of Mood States, and State-Trait Anxiety Inventory were used to measure psychological responses. We observed that walking in an urban park resulted in a significantly lower heart rate, higher parasympathetic nerve activity, and lower sympathetic nerve activity than walking through the city area. In subjective evaluations, participants were more “comfortable,” “natural,” “relaxed,” and “vigorous” after a walk in the urban park. Furthermore, they exhibited significantly lower levels of negative emotions and anxiety. These findings provide scientific evidence for the physiological and psychological relaxation effects of walking in urban parks during fall.

## 1. Introduction

Nowadays, most people live in cities, and this trend will likely continue in the future [1]. Urbanization has improved living conditions [2]. Moreover, it is a major factor in increasing life expectancies observed in many populations worldwide [1,3]. In addition, during health and nutrition improvements associated with urbanization, the burden of illness shifts from acute childhood infections to chronic and mostly non-communicable diseases, such as mental health disorders, diabetes, and cardiovascular disease, in adults [1,4,5].

However, from an evolutionary perspective, urbanization is a very drastic change that has occurred over a very short period. Rapid urbanization and artificial environments have caused environmental changes, such as increased traffic, polluted air and water, decreased agricultural land and natural open space [6], and anthropogenic climate change [7]. These changes threaten human health and quality of life [6,7]. Cities have been reported to have higher temperatures and thus act as urban heat islands [8,9,10] that are associated with sensations of discomfort and heat stress [11,12]. Exposure to outdoor urban air pollution is also associated with various adverse health outcomes, including heart disease, respiratory disease, and mortality [13]. Moreover, physical inactivity is recognized as a major problem because of increased sedentary behavior due to transport and office work [14,15].

Furthermore, the rapid spread of information technology in recent years has caused an increase in stress, which was referred to as “techno stress” in 1984 [16]. This is a modern disease of adaptation caused by an inability to cope with new computer technologies in a healthy manner. These stress is associated with poor psychological health [17,18], and many studies have reported the negative physiological impacts of stress on organisms, including humans [19,20,21].

In such a situation, attention has been focused on the role of urban green spaces in promoting human health and well-being. Urban green spaces can enhance the city environment by influencing temperature, wind, humidity, rainfall, soil erosion, flooding, air quality, visual quality, and sound quality, while also encouraging plant and animal diversity [22,23,24,25]. In addition, urban green space may provide important social and psychological benefits that enrich human life [26,27]. Recent demographic studies have found a positive association between exposure to urban green space and the perceived general health of residents [28,29,30]. Living in areas with accessible green spaces for walking also increased the longevity of senior citizens, independent of age, sex, marital status, baseline functional status, and socioeconomic status [30].

It is important to clarify physiological and psychological effects of urban green spaces. In a previous study, we examined the effects of walking in urban parks in spring [31] and winter [32] using the same experimental design and locations as in this study. Walking in an urban park improved positive mood and decreased negative mood and anxiety in both these seasons [31,32]. However, results of the physiological indices differed. A springtime walk in an urban park decreased heart rate, increased parasympathetic nerve activity, and suppressed sympathetic nerve activity compared with a walk in the city streets [31]. During low temperatures in winter, no significant difference in sympathetic nerve activity was detected between the two sites [32]. The effects of walking in urban parks were apparently different between the seasons. However, there is a lack of evidence-based research on the physiological and psychological effects of walking in urban green areas and the effects the seasons may have on these parameters. This study aimed to clarify the physiological and psychological effects of walking in urban parks during fall (autumn).

## 2. Experimental Section

### 2.1. Experimental Sites

**Figure 1:**
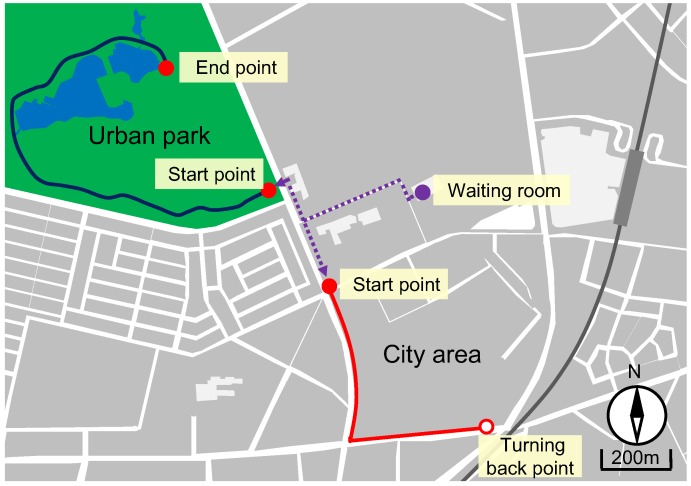
Experimental sites.

This field experiment was conducted on 7, 15, and 16 October 2014, in the Kashiwa-no-ha Park (hereafter denoted as the urban park) in Kashiwa City, Chiba Prefecture, Japan. A city area around the urban park (hereafter denoted as the city area) was selected as the control site. The urban park contained many hardwood trees, such as maple, tulip trees, cherry trees, and chestnut. Moreover, there was a large pond in the park center. As shown in Figure 1, in the urban park, the subjects walked around its large pond, whereas in the city area, they walked in the nearby residential area. Weather on the days of the experiment was sunny, except on October 15 when it was drizzling. The temperature, relative humidity, and intensity of illumination of the two experimental sites where the subjects walked (at 9–11 am and 1–3 pm) are shown in Table 1.

**Table 1:** Physical variables measured in the urban park and city area.

	Value (Mean ± SD)		P Value
	Urban Park	City Area	
Temperature (°C)	18.0 ± 1.7	19.2 ± 1.9	0.258
Relative humidity (%)	71.5 ± 10.1	64.7 ± 8.7	0.244
Intensity of illumination (lx)	24,230 ± 11,220	38,870 ± 22,330	0.192

P value by unpaired *t*-test.

### 2.2. Participants

Twenty-three Japanese male university students (mean age: 22.3 ± 1.2 years; Table 2) participated in this experiment. Participants were informed of the study’s aims and procedures prior to conducting the experiment; informed consent was also obtained. Alcohol, tobacco, and caffeine consumption was prohibited during the study period. The study was conducted in accordance with the Declaration of Helsinki, and the protocol was approved by the Ethics Committee of the Center for Environment, Health, and Field Sciences, Chiba University, Japan (Project identification code number: 5).

**Table 2:** Participant demographics.

Parameter	Value (Mean ± SD)
Total sample size	23
Sex	Male
Age (years)	22.3 ± 1.2
Height (cm)	171.1 ± 4.7
Weight (kg)	63.4 ± 8.1
BMI (kg/m^2^)	21.5 ± 2.1

### 2.3. Experimental Design

**Figure 2:**
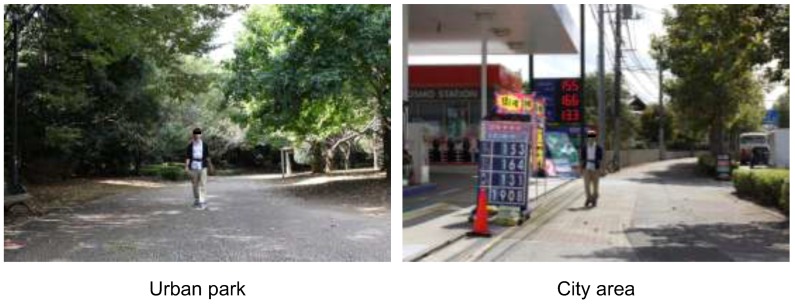
Experimental scenes.

Each subject walked in the urban park or city area for 15 min (Figure 2). We performed a within-subject experiment; two subjects were paired to eliminate the effect of the order of the sites walked. One subject walked in the urban park first and in the city area later, whereas the other walked in the city area first followed by the urban park. After walking, the subjects returned to the waiting room and completed the questionnaires. They rested for approximately 20 min and repeated the experiment in the reverse order. There were no significant differences in average walking speed between the two environments.

### 2.4. Physiological Indices

Heart rate and heart rate variability (HRV) were measured to assess autonomic nerve activity. HRV was measured using a portable electrocardiograph (Activtracer AC-301A, GMS, Tokyo, Japan); frequency spectra were generated using a HRV software tool (MemCalc/Win, GMS). For real-time HRV analysis using the maximum entropy method, inter-beat (R-R) intervals were continuously measured. Here two broad HRV spectral components were calculated: low (LF; 0.04–0.15 Hz) and high frequencies (HF; 0.15–0.40 Hz). HF component is an estimate of parasympathetic nerve activity, whereas LF/HF ratio is an estimate of sympathetic nerve activity [33,34]. To normalize HRV parameters across the subjects, we used natural logarithmic transformed values for the analysis [35]. Heart rate and HRV data were collected at 1-min intervals for each experimental location; the 15-min average was compared between sites.

### 2.5. Psychological Indices

Three different questionnaires were used to investigate the psychological responses after walking at each site. The semantic differential (SD) method [36] used three adjective pairs on seven scales, including “comfortable to uncomfortable”, “natural to artificial”, and “relaxed to awakening”. Profile of Mood State (POMS) [37,38,39] scores were determined for the following six sub-scales: “tension–anxiety”, “depression–dejection”, “anger–hostility”, “fatigue”, “confusion”, and “vigor”. A short form of POMS was used. State-Trait Anxiety Inventory (STAI) [40,41] was used to evaluate anxiety.

### 2.6. Statistical Analyses

Physiological data were used from 20 participants (data from the remaining three participants were excluded because of data collection errors). A paired *t*-test was used to compare mean physiological parameters between the two sites. Wilcoxon signed-rank test was used to analyze differences in the psychological indices reported after walking in the two environments. All statistical analyses were performed using SPSS 20.0 (IBM Corporation, Armonk, NY, USA). Statistical significance was fixed at *p* < 0.05. A one-side test was used in this study on the basis of the hypothesis that humans would also be relaxed by walking in urban parks during fall because it was found that nature-derived stimulation confers physiological and psychological relaxation effects, as reported in our previous research [31,32,42,43,44,45,46,47,48,49,50].

## 3. Results

We confirmed that there were no significant differences in the walking speed between the two environments (urban park: 4.11 km/h; city area: 4.07 km/h; *p* > 0.05), and energy expenditure in the urban park and the city area was 2.67 kcal/min and 2.82 kcal/min, respectively. Furthermore, before the walk, the baseline of physiological indices did not significantly differ between the two areas.

**Figure 3:**
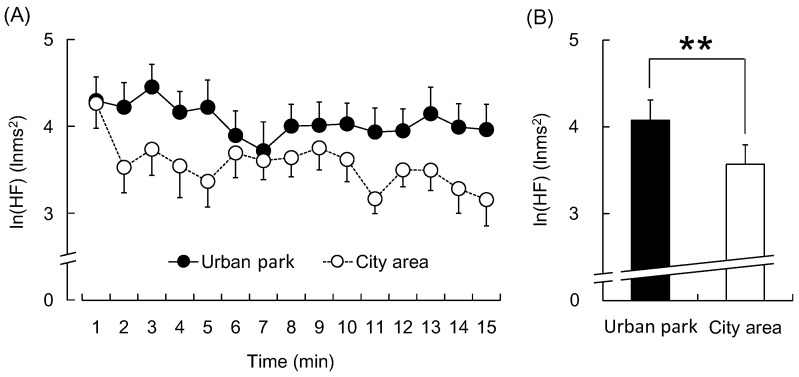
The 1-min averages and the overall mean ln(HF) value of heart rate variability during the urban park walk and the city area walk. (**A**) Change in each 1-min ln(HF) value; (**B**) Overall mean ln(HF) values. *N* = 20, mean ± standard error. ** *p* < 0.01, determined by the paired *t*-test (one-sided).

**Figure 4:**
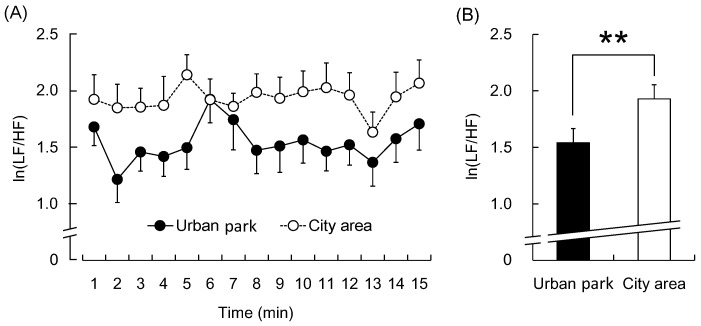
The 1-min averages and the overall mean ln(LF/HF) value of heart rate variability during the urban park walk and the city area walk. (**A**) Change in each 1-min ln(LF/HF) value; (**B**) Overall mean ln(LF/HF) values. *N* = 20, mean ± standard error. ** *p* < 0.01, determined by the paired *t*-test (one sided).

The participants exhibited statistically significant differences in their physiological and psychological responses to the 15-min walk in the urban park and city areas. Figure 3 shows the natural logarithm of the HF component ln(HF), which is an estimate of the parasympathetic nerve activity. In the 1-min segment analysis, all ln(HF) values were higher when participants walked in the urban park than when they walked in the city area (Figure 3A). The mean ln(HF) over the entire walking period was significantly higher in the urban park walking than in the city area walking (urban park: 4.1 ± 0.2 lnms^2^; city area: 3.6 ± 0.2 lnms^2^; *p* < 0.01, Figure 3B). In contrast, the natural logarithm of LF/HF (ln(LF/HF)), an estimate of sympathetic nerve activity, was lower during the urban park walk in the 1-min segment analysis (Figure 4A), whereas the average ln(LF/HF) over 15 min was significantly lower than that during the city walk (urban park: 1.54 ± 0.13; city area: 1.93 ± 0.13; *p* < 0.01, Figure 4B).

**Figure 5:**
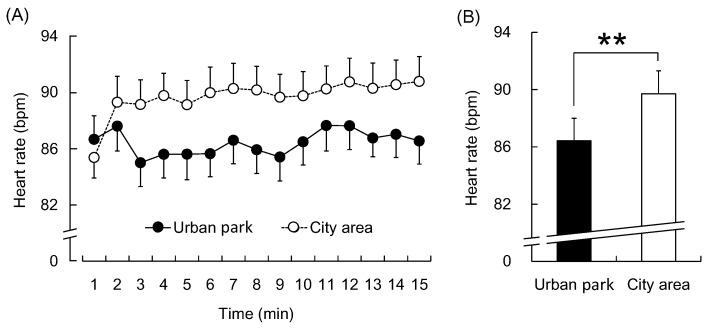
The 1-min averages and the overall mean heart rate during the urban park walk and the city area walk. (**A**) Changes in each 1-min average heart rate over the 15-min walk; (**B**) Overall mean heart rates. *N* = 20, mean ± standard error. ** *p* < 0.01, determined by the paired *t*-test (one-sided).

In addition, mean heart rate values within the 1-min segments were lower during the urban park walk than during the city area walk, except for the period of 1 min (Figure 5A). The mean heart rate over the entire 15-min period was significantly lower during the urban park walk than during the city walk (urban park: 86.4 ± 1.6 bpm; city area: 89.7 ± 1.6 bpm; *p* < 0.01, Figure 5B).

This study also included data for five subjects who walked on a rainy day. We examined additional data from these subjects. Compared with the city area walk, the mean ln(HF) over the entire walking period was marginally significantly enhanced (urban park: 4.2 ± 0.6 lnms^2^; city area: 3.9 ± 0.6 lnms^2^; *p* = 0.082), and the heart rate was marginally significantly reduced (urban park: 88.0. ± 2.4 bpm; city area: 90.0 ± 2.7 bpm; *p* = 0.066) during the brief walk in the urban park. Although there was no significant difference in ln(LF/HF).

**Figure 6:**
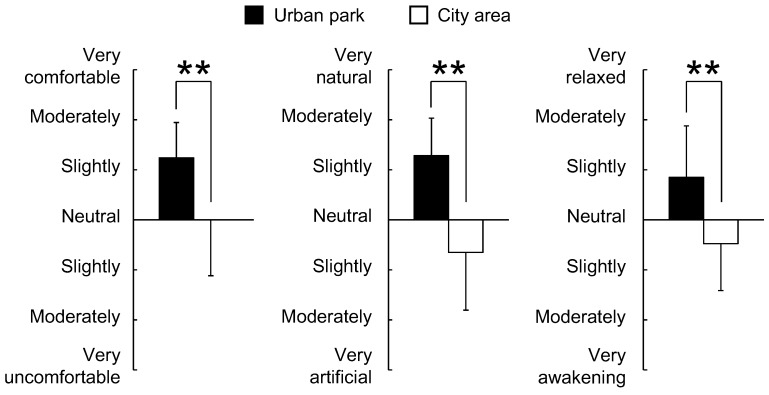
Comparison of subjective scoring for “comfortable”, “natural”, and “relaxed” feelings between the two environments according to the semantic differential method. *N* = 23, mean ± standard deviation. ** *p* < 0.01, determined by the Wilcoxon signed-rank test (one-sided).

**Figure 7:**
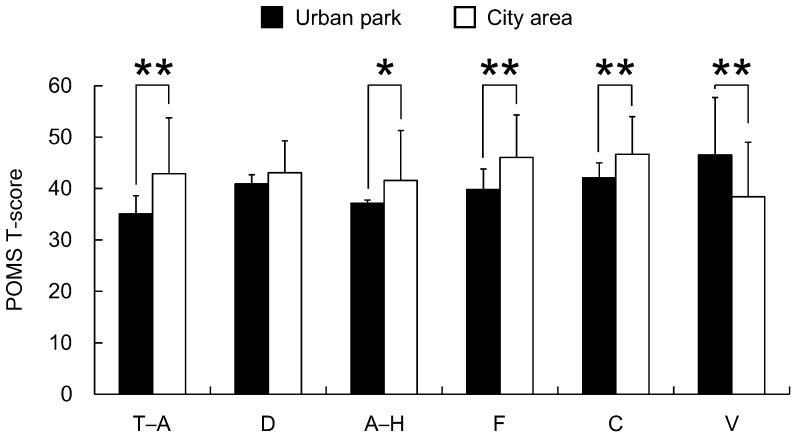
Comparison of subjective Profile of Mood State (POMS) scores between the two environments. T–A, tension–anxiety; D, depression–dejection; A–H, anger–hostility; F, fatigue; C, confusion; V, vigor. *N* = 23, mean ± standard deviation. * *p* < 0.05, ** *p* < 0.01, determined by the Wilcoxon signed-rank test (one-sided).

**Figure 8:**
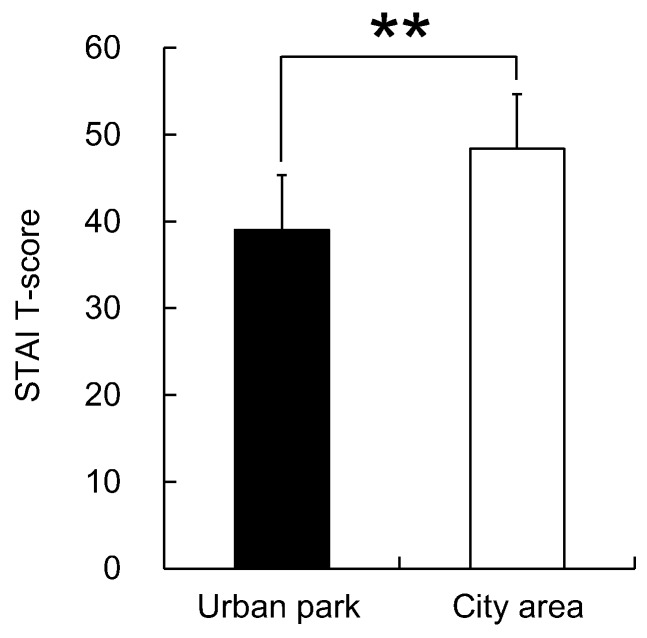
Comparison of subjective State-Trait Anxiety Inventory (STAI) scores between the two environments. *N* = 23, mean ± standard deviation. ** *p* < 0.01, determined by the Wilcoxon signed-rank test (one sided).

The analysis of the responses to the three questionnaires completed after the urban park and city area walks, including the SD method, POMS scores, and STAI scores, revealed differences in psychological responses between the two environments. Significantly higher SD scores were observed following the urban park walk than those following the city area walk for the following three adjectives: “comfortable”, “natural”, and “relaxed” (*p* < 0.01, Figure 6). Differences were also detected in the POMS test with scores for the negative subscales of “tension–anxiety”, “anger–hostility”, “fatigue”, and “confusion” being significantly lower after walking in the urban park than after walking in the city area (*p* < 0.05, Figure 7). Conversely, the positive mood state “vigor” was significantly higher after the urban park walk (*p* < 0.01, Figure 7). The score of “depression-dejection” was marginally significantly lower after walking in the urban park than after walking in the city area (*p* < 0.10, Figure 7). Finally, the total STAI score was 19.3% significantly lower after the urban park walk than after the city area walk (urban park: 39.0 ± 6.3; city area: 48.4 ± 7.5; *p* < 0.01, Figure 8).

## 4. Discussion

The findings indicated that a 15-min walk in an urban park induced physiological relaxation. Compared with those after a brief walk in the city area, parasympathetic nerve activity was significantly enhanced, sympathetic nerve activity was significantly suppressed, and heart rate was significantly lower during a brief walk in the urban park. These data are partly in agreement with those from previous studies investigating physiological responses to urban park walks during spring and winter [31,32]. Furthermore, in previous studies, the relaxation effects of walking or being in nature forests relative to those in an urban area were shown. These studies reported decreased blood pressure [42,43,44,45,46] and pulse rate [42,43,44,45,46,47,48], suppressed sympathetic nerve activity [42,44,45,46,47,48,49], increased parasympathetic nerve activity [42,44,45,46,47,48,49], decreased salivary cortisol levels [43,44,45,46,47,48,49,50], and decreased cerebral blood flow in the prefrontal cortex [50]. Recently, it has also been demonstrated that brief walking in a nature setting decreases not only self-reported rumination but also neural activity in the subgenual prefrontal cortex that is linked to mental illness risk [51]. The results of this study reveal an increased parasympathetic nerve activity and decreased sympathetic nerve activity and heart rate, and these observations are in accordance with the previous results for forest and urban walking, suggesting that even small natural areas within a larger urban area can confer similar health benefits.

According to the three questionnaires, the subjects in this study felt more “comfortable”, “natural”, “relaxed”, and “vigorous” after a walk in the urban park. In addition, negative emotions and anxiety were significantly lower after the urban park walk. These results, which exhibit the psychological benefits of walking in an urban park, are partly consistent with previous findings [31,32,42,43,44,45,48,49,50]. In modern times, mental health problems associated with living in urban environment are profound. For example, McKenzie *et al.* reported that urban living environments are associated with higher rates of psychotropic medication prescriptions for anxiety, depression, and psychosis [52]. Therefore, the psychological benefits of walking in urban green space, (*i.e*., improved mood state and decreased anxiety level) are very significant, and urban green space is expected to play a very important role in the promotion of mental health in the future.

The beneficial effects of urban green space suggest a simple, accessible, and cost-effective method to improve the quality of life and health of urban residents. In a previous study, in addition to fall, we examined the effects of walking in the urban park in spring [31] and winter [32] using the same experimental design and locations. The current results corroborate our previous findings. However, in the comparisons of the three seasons, in winter, there was no significant difference detected in sympathetic nervous activity between the two sites, whereas all other parameters (parasympathetic nervous activity, heart rate, and psychological indices) demonstrated similar differences between the walking environments. We do not know the exact reason for the different results obtained for winter; however, we suppose they resulted from seasonal differences in environmental conditions, such as temperature (spring: 24.7 °C, fall: 18.0 °C, and winter: 13.8 °C), humidity (spring: 39.2%, fall: 71.5%, and winter: 50.9%), and wind speed as well as the state of trees, such as the color and quantity of leaves. This issue will need to be considered in more depth in the future after more data is accumulated.

This study also included data for five subjects who were out walking on a rainy day. Most previous studies relating to the physiological effect of the natural environment were conducted on a sunny day. To the best of our knowledge, there are no evidence-based research studies that clarify the physiological and psychological effects of walking in an urban park on a rainy day. Therefore, although the sample size is small, we examined these subjects who walked in an urban park and city area on a rainy day. As a result, similar results were obtained. Compared with the city area walk, parasympathetic nerve activity was marginally significantly enhanced (urban park: 4.2 ± 0.6 lnms^2^; city area: 3.9 ± 0.6 lnms^2^; *p* = 0.082), and heart rate was marginally significantly reduced (urban park: 88.0. ± 2.4 bpm; city area: 90.0 ± 2.7 bpm; *p* = 0.066) during a brief walk in the urban park. It has been observed that walking in an urban park confers an effect of physiological relaxation even on a rainy day.

These findings provide scientific evidence for the beneficial physiological and psychological effects of walking in urban parks during fall. However, this study had several limitations. First, to generalize the findings, further studies based on a larger sample (including various other demographic groups, such as females and different ages) are required. Second, for overall discussion, future studies should determine the effects of urban green space using other physiological indices, such as brain activity, autonomic nerve activity, and endocrine activity. Finally, the subjects’ prior expectations and experience with nature and nature relatedness may influence the results. All these limitations must be considered in future research.

## 5. Conclusions

These findings provide scientific evidence for the beneficial physiological and psychological effects of walking in urban parks during fall. A brief walk in an urban park can induce parasympathetic nerve activity, suppress sympathetic nerve activity, decrease the heart rate, enhance the mood state, and reduce anxiety. In conclusion, walking in urban parks confers physiological and psychological relaxation effects during fall.
